# Health-Related Quality of Life measured with EQ-5D-5L among tuberculosis patients in Addis Ababa, Ethiopia: Institutional-based cross-sectional study

**DOI:** 10.1371/journal.pone.0326033

**Published:** 2025-06-24

**Authors:** Tenaw Baye Tarekegn, Getachew Alemkere, Eskinder Eshetu Ali

**Affiliations:** 1 Department of Pharmacy, College of Health Sciences, Woldia University, Woldia, Ethiopia; 2 School of Pharmacy, College of Health Sciences, Addis Ababa University, Addis Ababa, Ethiopia; Arsi University College of Health Sciences, ETHIOPIA

## Abstract

**Background:**

Tuberculosis (TB) substantially compromises health-related quality of life (HRQoL), yet limited studies have assessed its impact on Ethiopian patients using the EQ-5D instrument. This study evaluates HRQoL, estimates health state utility values, and identifies associated factors among TB patients in Ethiopia.

**Methods:**

A cross-sectional study was conducted across 20 public health centers in Addis Ababa, Ethiopia, involving 672 TB patients selected via proportional allocation. HRQoL was measured using the EuroQol five-dimension five-level (EQ-5D-5L) tool. Predictors of utility scores were analyzed using the Kruskal-Wallis test and Tobit censored regression models.

**Results:**

Anxiety/depression was the most frequently affected dimension (55.4% of participants). The mean EQ-5D-5L utility score was 0.91 (SD ± 0.14), and the mean EQ-VAS score was 80.6 (SD ± 15.6). Older age (55–64 years: β = −0.067, *p* < 0.001; ≥ 65 years: β = −0.383, *p* < 0.001) and unemployment (β = −0.119, *p* < 0.001) were associated with significantly lower HRQoL. Conversely, higher income (β = 0.056, *p* < 0.001), absence of comorbidities (β = 0.059, *p* < 0.001), and mid-treatment duration (4–5 months: β = 0.029, *p* = 0.011) correlated with better HRQoL.

**Conclusion:**

The findings underscore the influence of socio-demographic and clinical factors on HRQoL among TB patients in Ethiopia, calling for targeted interventions and policy reforms to enhance treatment outcomes and patient support.

## Introduction

Tuberculosis (TB) remains the world’s deadliest infectious disease, with 7.5 million new cases and 1.3 million deaths reported globally in 2022 [1]. The African region bears a disproportionate burden, accounting for 23% of cases, with Ethiopia ranking among the 14 high-burden countries for TB, multidrug-resistant TB (MDR-TB), and TB-HIV coinfection [1]. In 2021, Ethiopia reported 157,000 TB cases and a mortality rate of 24 deaths per 100,000 population—representing 2.4% of global TB-related deaths [2]. Beyond its clinical manifestations, TB imposes severe socioeconomic consequences, including stigma, financial hardship, and marked reductions in health-related quality of life (HRQoL) [1,3].

The World Health Organization’s End TB Strategy prioritizes patient-centered care, underscoring HRQoL assessment as a key treatment outcome metric. Studies consistently show that TB reduces HRQoL scores by 35–50% across measurement tools [1,2]. Regional studies demonstrate substantial HRQoL impairments among TB patients, with variations across clinical and socioeconomic contexts. South African study exhibit utility scores of 0.62 (versus 0.85 in healthy controls) [4], while Zimbabwean studies highlight socioeconomic gradients where employed, educated patients maintain higher utilities [5]. A Thai cohort study revealed age, monthly income and HIV co-infection as independent predictors of EQ-5D [6]. A meta-analysis of 27 studies [7] further confirms pronounced HRQoL deficits in low-income settings. Despite Ethiopia’s status as one of highest-burden African country, EQ-5D-5L-based HRQoL data remain absent, a critical gap hindering targeted interventions and economic evaluations of TB programs.

The EuroQol 5-Dimension 5-Level (EQ-5D-5L) instrument is increasingly adopted in TB research due to its psychometric robustness and cross-cultural validity [7–9]. Compared to alternatives like the 36-Item Short Form Health Survey (SF-36), World Health Organization Quality of Life – Brief Version (WHOQOL-BREF), and 30-Item General Health Questionnaire (GHQ-30), the EQ-5D-5L offers distinct advantages: superior sensitivity to clinical changes and reduced ceiling effects (demonstrated in Vietnamese cohorts) [10]; enhanced capacity to capture severe disability in advanced TB; and utility valuation for cost-effectiveness analyses [11]. Its five-level response scale also improves precision over the three-level EQ-5D-3L [12].

This study aims to establish baseline EQ-5D-5L utility values for Ethiopian TB patients, identify modifiable HRQoL determinants, and generate data to inform disability-adjusted life year (DALY) calculations and economic evaluations of TB interventions in Ethiopia.

## Methods

### Study setting and design

A cross-sectional study was conducted across public health centers in Addis Ababa, Ethiopia, from December 2021 to March 2022. Addis Ababa—the capital and most populous city in Ethiopia had an estimated population of 3,770,554 (2020/2021 Ethiopian Fiscal Year) [13]. The city comprises ten administrative sub-cities and houses 117 public health centers alongside 15 governmental hospitals. Due to high population density and living conditions conducive to transmission, Addis Ababa reports a substantial TB burden, with 7,027 bacteriologically confirmed new TB cases documented in 2020/2021 [13]. The study setting was selected based on: high TB prevalence, reflecting urban transmission dynamics; comprehensive DOTS coverage, with all 117 health centers providing standardized TB treatment; and geographic diversity, ensuring representative sampling across sub-cities. Health centers were stratified by sub-city, with two randomly selected from each (total *n* = 20). This approach ensured proportional representation of Addis Ababa’s heterogeneous population.

### Ethical considerations

Ethical approval was obtained from: the Ethical Review Committee of the School of Pharmacy, Addis Ababa University (ERB/SOP/320/13/2021); and the Addis Ababa Regional Health Bureau (A/A/T/3284/227). All participants provided written informed consent after receiving a detailed explanation of the study objectives. Confidentiality was maintained by excluding personal identifiers from data collection forms, and consent included permission to publish anonymized responses. The study adhered to the principles of the Declaration of Helsinki.

### Recruitment of participants

The study enrolled adult TB patients (≥18 years) receiving treatment at participating health centers during the study period. Exclusion criteria included: documented cognitive or psychiatric conditions impairing informed consent or interview participation; and severe hearing impairment preventing reliable questionnaire administration. Recruitment continued at each site until predetermined sample size targets were achieved, ensuring proportional representation across all study locations.

### Sample size determination and sampling technique

The minimum sample size was calculated using the single population proportion formula:


$$\begin{array}{c} n=Z²p(1-p)/d² \end{array}$$

Where:

- Z = 1.96 (95% confidence level).
- p = 0.5 (anticipated proportion of TB patients with better-than-average HRQoL).
- d = 0.05 (margin of error).

This yielded n = 384 for infinite populations. For our finite population (N = 2,227 expected TB cases during the study period), we applied the finite population correction:


$$\begin{array}{c} nf=\pi ni/(1\;+\;ni/N) \end{array}$$

Where:

-  nf = final sample size,
-  ni = sample size for more than 10,000 population, and
-  N = population size.

$\begin{array}{c} nf=384/(1+384/2227)=384/1.2=320 \end{array}$,

To account for the stratified sampling design across 20 health centers, we incorporated a design effect of 2 (n × design = 320 × 2 = 640). Adding a 5% buffer for non-response yielded the final sample size of 672 participants.

The sampling technique involved a two-stage process to ensure representative and efficient participant selection. First, 20 health centers were chosen through stratified random sampling by sub-city, ensuring each sub-city was adequately represented. The sample allocation was proportional to each center’s tuberculosis (TB) caseload, with varying numbers of participants selected from different centers (e.g., 31 from Addis Ketema, 50 from Addis Raey, etc.; see S1 Table). Within each selected health center, participants were consecutively enrolled until the predetermined center-specific quotas were met.

### Data collection instruments

Health-related quality of life (HRQoL) was assessed using the Amharic versions of the EQ-5D-5L and EQ-VAS questionnaires, which have been validated for Ethiopia’s general population [14]. The EQ-5D-5L instrument evaluates five health dimensions: mobility (MO), self-care (SC), usual activities (UA), pain/discomfort (PD), and anxiety/depression (AD), with each dimension rated across five severity levels (1 = no problems to 5 = extreme problems). This structure yields 3,125 (55) possible health states, ranging from 11111 (optimal health) to 55555 (worst health state). For analysis, responses were dichotomized into no problems (level 1) versus any problems (levels 2–5). The instrument generates a utility score anchored at 0 (death) to 1 (full health), calculated using country-specific value sets [15]. The EQ-VAS complemented this assessment through a 20-cm vertical visual analog scale (0 = worst imaginable health to 100 = best imaginable health), providing a patient-reported global health measure. Clinical data, including TB classification, treatment duration, and comorbidities, were extracted from medical records. Socio-demographic characteristics were collected using a structured questionnaire that underwent rigorous translation (English-to-Amharic) with back-translation to ensure conceptual equivalence. Medication-related questions were maintained in English to preserve technical accuracy. All instruments were administered by trained interviewers using the official Amharic translations provided by the EuroQol Group, with standardized protocols to ensure data consistency across study sites.

### Data collection procedure and quality assurance

Twenty nurse professionals from participating TB clinics were recruited as data collectors following a standardized one-day training program. Prior to the main study, we conducted a pretest involving 34 participants (5% of the target sample size) at a non-study health center, which informed final adjustments to the data collection instruments. Following written informed consent procedures, trained collectors administered face-to-face interviews to capture socio-demographic characteristics and HRQoL measures (EQ-5D-5L and EQ-VAS responses). Clinical data, including medication history and treatment parameters, were subsequently abstracted from medical records. All completed questionnaires underwent immediate on-site review for completeness and consistency before submission. We implemented rigorous quality control measures throughout the data management process, including double-entry verification and range checks during database development. The field data collection occurred over a focused period from 27 December 2021 to 3 February 2022, with daily supervisor oversight to maintain protocol adherence across all study sites.

### Operational definitions

#### Income classification.

Income levels were classified according to the World Bank Group’s thresholds for Ethiopia, with lower income defined as ≤1.90/day (approximately ≤57/month), middle income as 5.50/day (165/month), and higher income as 5.50–20/day (~165–600/month) [16].

#### No formal education.

The category “no formal education” was assigned to individuals without any structured institutional education, including those who had never attended school or university.

### Data analysis

Socio-demographic, clinical, and medication-related characteristics of participants were summarized using frequency distributions and percentages. EQ-5D-5L health profiles were similarly described. The Kolmogorov-Smirnov test revealed non-normal distribution of utility scores (evidenced by skewness and kurtosis), prompting presentation of both mean (standard deviation) and median (range) values for health status summary statistics. Initial assessment of group differences in utility scores employed Kruskal-Wallis tests for all variables potentially associated with HRQoL (socio-demographic, clinical, and medication-related factors). Variables demonstrating significant associations (p ≤ 0.05) were subsequently entered into multivariable Tobit regression models. This approach was selected to address characteristic ceiling effects in EQ-5D data [17–22] and accommodate the study’s utility score range (−0.10 to 1.0), which required censored regression techniques for proper handling of left- and right-censored observations. Utility score calculations incorporated a hybrid regression model specifically adapted for Ethiopia’s general population [14] implemented using level-specific utility value factors. The scoring algorithm was executed in Excel 2016 to generate final utility values (Equation 1).


$$\begin{array}{cc} Utilityvalue= & 1-(mo2*(0.0337)+mo3*(0.0644)+mo4*(0.2276)+mo5*(0.3598)\\ & +sc2*(0.0235)+sc3*(0.0395)+sc4*(0.1419)+sc5*(0.2223)\\ & +ua2*(0.0323)+ua3*(0.0483)+ua4*(0.1574)\\ & +ua5*(0.2721)+pd2*(0.0361)+pd3*(0.0516)+pd4*(0.2703)\\ & +pd5*(0.4064)+ad2*(0.0259)+ad3*(0.0848)+ad4*(0.2987)\\ & +ad5*(0.4578)). \end{array}$$

Where; mo = mobility, sc = self-care, ua = usual activity, Pd = pain/discomfort, ad = Anxiety/ depression.

Eq. 1: The equation for computing the utility score

STATA software version 17 was utilized for all statistical analyses, with statistical significance set at p ≤ 0.05.

## Results

### Socio-demographic and clinical characteristics

The study enrolled 672 TB patients, with a male predominance (54.3%) and mean age of 38.5 years (±1.46 SD; range: 18–80 years). Most participants (61.4%) belonged to the 25–54 age group. Nearly all (96%) had received formal education, while 50.1% were married and 62.5% employed. HIV co-infection was present in 12.0% of cases. Pulmonary TB accounted for 71.7% of cases (n = 482), including 367 bacteriologically confirmed cases. New TB diagnoses predominated (91.1%, n = 612), with a mean treatment duration of 3.31 months (±1.91 SD). First-line anti-TB medications were used by 95.5% of patients (n = 642) (Table 1).

**Table 1:** Socio-demographic, clinical characteristics, and reported health problems among tuberculosis patients in public health centers of Addis Ababa, Ethiopia, 2022 (n = 672).

Study variables	Category	N (%)	% reporting problems				
			Mobility	Self-care	Usual-activities	Pain/discomfort	Anxiety/depression
	Total participants	672 (100)	9.9	10	25.9	52.7	55.4
Gender	Male	365 (54.3)	8.8	9.3	23.5	51.8	56.2
	Female	307 (45.7)	10.7	9.0	28.7	54.7	54.4
Age (years)	Age (mean, SD)	38.5 (±1.46)					
	18-24	160 (23.8)	4.4	3.7	13.1	36.2	41.9
	25-54	413 (61.4)	6.5	7.3	23.2	48.7	51.0
	55-64	65 (9.7)	12.3	9.2	40.0	95.4	93.8
	≥ 65	34 (5.1)	67.6	73.5	91.2	97.0	97.0
Marital status	Single	274 (40.8)	6.9	7.3	24.8	47.4	52.2
	Married	337 (50.1)	8.0	7.1	21.0	54.3	53.7
	Divorced	30 (4.5)	6.7	13.3	33.3	53.3	73.3
	Widowed	31 (4.6)	54.8	61.3	80.6	80.6	84.0
Educational status	No formal education	27 (4.0)	33.3	30.0	51.8	59.2	66.7
	Primary education (1–8)	141 (21.0)	17.7	16.3	33.3	59.6	54.6
	Secondary education (9–12)	262 (39.0)	7.2	7.6	23.6	55.3	59.9
	Higher education	242 (36.0)	4.9	6.2	21.0	45.0	49.6
Habitation	Urban	651 (96.9)	9.0	9.5	25.3	52.4	55.3
	Rural	21 (3.1)	28.6	23.8	42.8	61.9	57.1
Occupational status	Employed (Government or Private organization)	183 (27.2)	2.7	3.3	14.7	38.2	45.3
	Self-employed (Private business)	237 (35.3)	6.3	6.3	24.5	51.5	52.7
	Unemployed	43 (6.4)	41.9	41.9	74.4	81.4	86.0
	Retiree	24 (3.6)	37.5	41.6	66.7	70.8	70.8
	Student	65 (9.7)	4.6	6.1	16.9	47.7	53.8
	Housewife	93 (13.8)	14.0	11.8	25.8	64.5	61.3
	Daily laborer	27 (4.0)	7.4	11.1	22.2	70.4	66.7
Household monthly income	Low (<3000)	233 (34.7)	18.9	18.4	41.2	72.9	74.7
	Middle (3000–9000)	380 (56.5)	5.5	6.3	20.3	47.6	50.5
	High (>9000)	59 (8.8)	–	–	1.7	5	10.2
Co-morbidities	HIV infection	81 (12)	13.6	11.1	29.6	79.0	77.8
	Others*	29 (4.4)	41.4	37.9	44.8	58.6	68.9
	None	562 (83.6)	7.5	8.4	24.4	48.6	51.4
Drug susceptibility test	Yes	326 (48.5)	9.2	9.2	23.6	54.0	57.4
	No	346 (51.5)	10.1	10.7	28.0	51.4	53.5
Method of drug administration	Directly observed treatment (DOT)	122 (18.2)	16.4	12.3	32.0	58.2	60.6
	Self-administered	550 (81.8)	33.6	9.4	24.5	51.4	54.2
Length of time since treatment initiation	<2 month	126 (18.8)	26.2	27.0	51.6	84.1	90.5
	2-3 month	257 (38.2)	8.2	8.9	24.9	55.2	54.5
	4-5 month	215 (32.0)	0.9	0.9	15.3	36.3	40.5
	>5 month	74 (11)	12.2	10.8	16.2	37.8	41.9
TB type	Pulmonary positive Pulmonary negative	367 (54.6) 115 (17.1)	7.9 8.9	7.6 9.5	23.4 21.7	51.5 49.5	55.8 54.8
	Extra pulmonary	190 (28.3)	13.7	14.7	32.6	56.3	54.2
TB class	New	612 (91.1)	9.3	9.8	25.8	51.8	54.4
	Relapse	60 (8.9)	13.3	11.7	26.7	61.7	65.0
Type of medication	First line	642 (95.5)	9.5	9.5	24.4	52.0	55.0
	Second line	30 (4.5)	13.3	23.4	56.7	66.7	63.3

*others: Hypertension, Diabetes mellitus, asthma, and kidney diseases; HIV: Human Immune Virus; TB: Tuberculosis.

### Health-related quality of life profile

**Fig 1:**
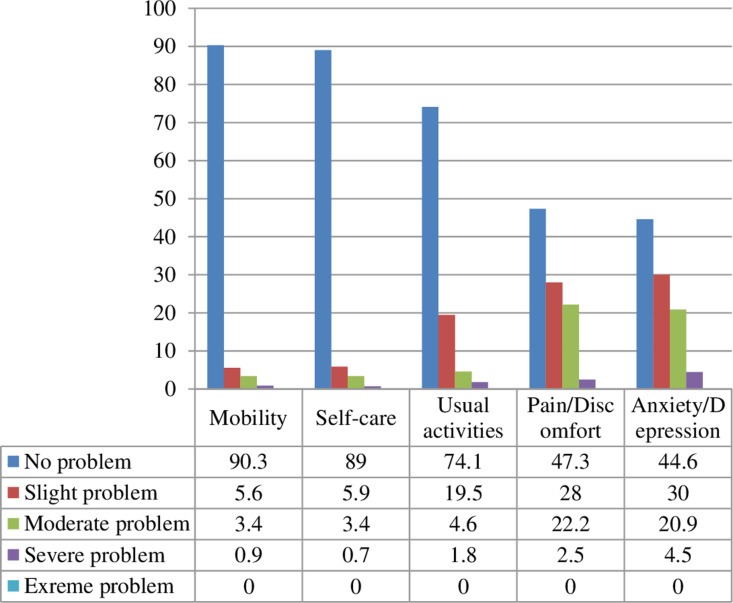
Percentage distribution of participants to the five domains of the EQ-5D-5L.

The EQ-5D-5L assessment revealed anxiety/depression as the most impaired dimension, with 55.4% of participants reporting any level of problems (30.0% slight, 4.5% severe). Pain/discomfort affected 28.0% of respondents. In contrast, most patients reported no limitations in mobility (90.3%), self-care (89.0%), or usual activities (74.1%). No extreme problems (level 5) were reported in any dimension. While 34.5% of patients (n = 232) achieved a “perfect health state” (11111) on the EQ-5D-5L descriptive system, only 12.2% (n = 82) attained the maximum 100-point score on the EQ-VAS. Notably, no participants reported the worst possible health states (55555 on EQ-5D-5L or 0 on EQ-VAS) (Fig 1).

### Stratified health-related quality of life outcomes

Sex-based analysis revealed gender disparities in reported problems: female participants showed higher impairment proportions in mobility (10.7% vs males), usual activities (28.7%), and pain/discomfort (54.7%), whereas males reported greater anxiety/depression symptoms (56.2%). Age demonstrated a strong positive association with problem frequency, with participants aged >65 years exhibiting the highest overall impairment (42.2% across all dimensions, n = 70/166). This age-related gradient was consistent across all five EQ-5D-5L dimensions. Socioeconomically disadvantaged groups – including those with no formal education, rural residents, widowed individuals, low-income earners (≤$57/month), and unemployed/retired participants – consistently reported higher problem frequencies in all dimensions compared to their counterparts (Table 1).

Clinical factors further differentiated outcomes: TB-HIV co-infected patients showed particularly elevated impairment in pain/discomfort (79.0%) and anxiety/depression (77.8%). Comorbid conditions (e.g., diabetes mellitus, heart failure, asthma) were associated with marked limitations in usual activities (44.8% impairment). Treatment-related vulnerabilities emerged among patients with: [1] early treatment phase (<2 months duration), [2] extra-pulmonary TB diagnoses, [3] relapse cases, and [4] second-line regimens – all demonstrating elevated problem frequencies across dimensions (Table 1).

### EQ-5D-5L utility and EQ-VAS scores

**Fig 2:**
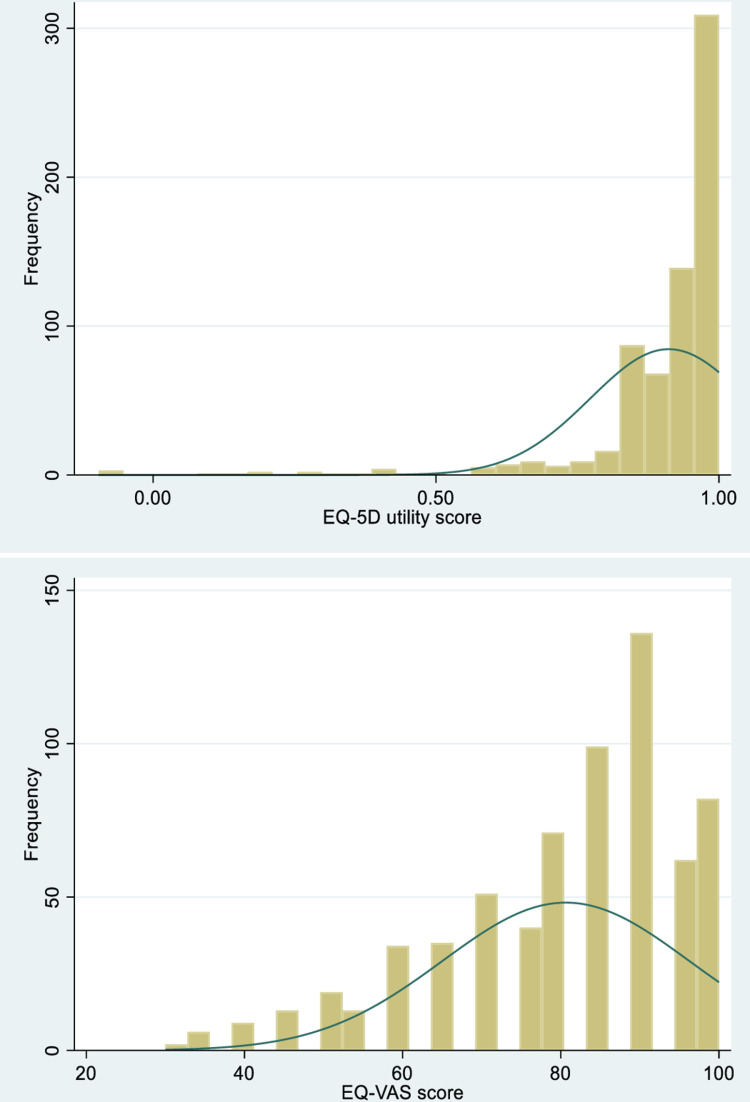
Distribution of EQ-5D-5L utility and EQ-VAS scores among tuberculosis patients.

The study population demonstrated moderately high health-related quality of life scores, with a mean EQ-5D-5L utility index of 0.91 (SD ± 0.14) and a mean EQ-VAS score of 80.6 (SD ± 15.6). Both scoring distributions exhibited significant right-skewness, indicating a concentration of responses toward the upper end of the scales (Fig 2). The utility scores ranged from −0.10 to 1.00, with 34.5% of participants (n = 232) achieving the maximum utility score of 1.00 (perfect health). Similarly, EQ-VAS scores showed ceiling effects, with 12.2% (n = 82) reporting the highest possible rating of 100. Notably, the distribution patterns revealed that while most participants clustered toward favorable health states, a small but clinically important subgroup reported substantially impaired scores, particularly in the lower quartiles of both measures.

### Determinants of health-related quality of life in tuberculosis patients

Initial non-parametric analysis using the Kruskal-Wallis test identified several significant associations with reduced HRQoL. Older patients demonstrated markedly lower scores, with those ≥65 years showing a mean utility of 0.51 compared to younger cohorts (p < 0.01). Employment status and education level similarly influenced outcomes, as unemployed individuals reported significantly impaired HRQoL (mean utility 0.71) and those without formal education scored lower (0.85) than their more educated counterparts (0.93, p < 0.01). Clinical factors including HIV co-infection reduced scores by an average of 0.07 utility points (p < 0.001), while treatment duration showed a positive association, with scores improving from 0.82 (<2 months treatment) to 0.95 (>4 months treatment, p < 0.001). Notably, neither gender nor TB type showed significant associations with HRQoL (p > 0.05) (Table 2).

**Table 2:** EuroQol five-dimension five-level (EQ-5D-5L) utility scores across participant characteristics in public health centers of Addis Ababa, Ethiopia, 2022 (n = 672).

Variables		N	Mean ± SD	Median (range)	p-value
Gender	Male	365	0.91 ± 0.14	0.93 (−0.10-1)	0.177
	Female	307	0.90 ± 0.13	0.93 (−0.10-1)	
Age (years)	18-24	160	0.95 ± 0.07	0.97 (0.29-1)	<0.001*
	25-54	413	0.93 ± 0.09	0.94 (−0.10-1)	
	55-64	65	0.86 ± 0.06	0.86 (0.62-0.97)	
	≥ 65	34	0.51 ± 0.25	0.60 (−0.10-0.81)	
Marital status	Married	337	0.91 ± 0.11	0.93 (−0.10-1)	0.829
	Single, divorced, widowed	335	0.90 ± 0.16	0.93 (−0.10-1)	
Educational status	No formal education	27	0.85 ± 0.21	0.91 (−0.10-1)	**0.003** ^*^
	Primary education (1–8)	141	0.88 ± 0.18	0.93 (−0.10-1)	
	Secondary education (9–12)	262	0.90 ± 0.12	0.93 (0.19-1)	
	Higher education	242	0.93 ± 0.10	0.97 (0.12-1)	
Habitation	Urban	651	0.91 ± 0.13	0.93 (−0.10-1)	0.149
	Rural	21	0.81 ± 0.31	0.91 (−0.10-1)	
Occupational status	Employed	183	0.94 ± 0.08	0.97 (0.40-1)	<0.001*
	Private business	237	0.92 ± 0.10	0.93 (0.34-1)	
	Student	65	0.93 ± 0.10	0.96 (0.29-1)	
	Housewife	93	0.90 ± 0.12	0.93 (0.20-1)	
	Daily laborer	27	0.91 ± 0.08	0.93 (0.66-1)	
	Unemployed	43	0.71 ± 0.31	0.84 (−0.10-1)	
	Retiree	24	0.84 ± 0.16	0.86 (0.19-1)	
Household monthly income in ETB	Low (<3000)	233	0.86 ± 0.17	0.91 (−0.10-1)	0.001*
	Middle (3000–9000)	380	0.92 ± 0.11	0.96 (0.13-1)	
	High (>9000)	59	0.99 ± 0.01	1.00 (0.92-1)	
Co-morbidities	HIV infection	81	0.85 ± 0.18	0.88 (−0.10-1)	<0.001*
	Others**	29	0.88 ± 0.19	0.90 (−0.10-1)	
	None	562	0.92 ± 0.12	0.95 (−0.10-1)	
Drug susceptibility test	Yes	326	0.90 ± 0.14	0.93 (−0.10-1)	0.744
	No	346	0.91 ± 0.13	0.93 (−0.10-1)	
Method of drug administration	DOT	122	0.89 ± 0.14	0.93 (0.13-1)	0.038*
	Self-administered	550	0.91 ± 0.14	0.94 (−10−1)	
Length of time since treatment initiation	<2 month	126	0.82 ± 0.18	0.86 (−0.10-1)	<0.001*
	2-3 month	257	0.91 ± 0.14	0.93 (−0.10-1)	
	4-5 month	215	0.95 ± 0.08	0.97 (0.12-1)	
	>5 month	74	0.93 ± 0.10	0.99 (0.34-1)	
TB type	Pulmonary positive	367	0.92 ± 0.13	0.94 (−0.10-1)	0.299
	Pulmonary negative	115	0.91 ± 0.13	0.94 (0.13-1)	
	Extra pulmonary	190	0.89 ± 0.15	0.93 (−0.10-1)	
TB class	New	612	0.91 ± 0.13	0.93 (−0.10-1)	0.152
	Relapse	60	0.88 ± 0,16	0.93 (0.28-1)	
Type of medication	First line	642	0.91 ± 0.14	0.93 (−0.10-1)	0.061
	Second line	30	0.87 ± 0.14	0.93 (0.40-1)	
Total		672	0.91 ± 0.14	0.93 (−0.10; 1)	

Statistical analysis: non-parametric Kruskal–Wallis rank test; *p < 0.05; ** others: Hypertension, Diabetes mellitus, asthma, and kidney diseases; DOT: Direct Observable Therapy, ETB: Ethiopian Birr, HIV: Human Immune Virus, TB: Tuberculosis.

Multivariable Tobit regression analysis, controlling for potential confounding factors, confirmed several key determinants while refining our understanding of their relative contributions. Age maintained a strong inverse relationship with HRQoL, with patients aged 55–64 years showing significantly reduced scores (β = −0.067, 95% CI = −0.096 to −0.037; p < 0.001) and those >65 years demonstrating even greater impairment (β = −0.383, 95% CI = −0.421 to −0.345; p < 0.001). Unemployment remained independently associated with poorer outcomes (β = −0.119, 95% CI = −0.155 to −0.083; p < 0.001). Protective factors included higher household income (β = 0.056, 95% CI = 0.027 to 0.085; p < 0.001), absence of comorbidities (β = 0.059, 95% CI = 0.036 to 0.081; p < 0.001), and longer treatment duration (4–5 months: β = 0.029, 95% CI = 0.007 to 0.052; p = 0.011). Interestingly, while educational status showed significance in univariate analysis, it did not retain independent predictive value in the adjusted model, suggesting its effects may be mediated through other socioeconomic factors. Similarly, method of drug administration showed no significant association in the multivariable analysis (Table 3).

**Table 3:** Tobit regression model results for EQ-5D-5L utility scores among tuberculosis patients in public health centers of Addis Ababa, Ethiopia, 2022 (n = 672).

Patient characteristics		EQ-5D index		
		Coef.	95% CI	p-value
Age (years)	18-24 (a)			
	25-54	−0.017	−0.037; 0.001	0.068
	55-64	−0.067	−0.096; −0.037	0.000***
	≥ 65	−0.383	−0.421; −0.345	0.000***
Educational status	No formal education (a)			
	Primary education (1–8)	0.021	−0.018; 0.061	0.293
	Secondary education (9–12)	0.010	−0.029; 0.050	0.601
	Higher education	0.011	−0.030; 0.053	0.590
Occupational status	Employed (a)			
	Private business	−0.013	−0.034; 0.008	0.239
	Student	−0.027	−0.059; 0.004	0.089
	Housewife	−0.015	−0.042; 0.013	0.293
	Daily laborer	−0.034	−0.075;.007	0.101
	Retiree	−0.029	−0.070; 0.013	0.178
	Unemployed	−0.119	−0.155; −0.083	0.000***
Household monthly income in ETB	Low (<3000) (a)			
	Middle (3000–9000)	0.014	−0.002; 0.031	0.087
	High (>9000)	0.056	0.027; 0.085	0.000***
Co-morbidities	HIV infection (a)			
	Others*^a^	0.055	0.014; 0.095	0.008**
	None	0.059	0.036; 0.081	0.000***
Method of drug administration	DOT (a)			
	Self-administered	0.002	−0.017; 0.020	0.858
The length of time since treatment initiation	<2 month (a)			
	2-3 month	0.007	−0.014; 0.029	0.502
	4-5 month	0.029	0.007; 0.052	0.011*
	>5 months	0.024	−0.005; 0.052	0.104

***p < 0.001, **p < 0.01, *p < 0.05, ^a^Reference Group; *others: Hypertension, Diabetes mellitus, asthma, and kidney diseases; ETB: Ethiopian Birr, HIV: Human Immune Virus.

## Discussion

This study represents the most comprehensive assessment to date of HRQoL and associated factors among TB patients in Ethiopia using the validated EQ-5D-5L instrument. The findings reveal a complex picture of the TB experience in Ethiopia, revealing both significant health burdens and important contextual factors that moderate these impacts. The most notable finding is the profound mental health burden of TB, with 55.4% of participants reporting anxiety/depression symptoms and 52.7% experiencing pain/discomfort. These rates substantially exceed those reported in the general Ethiopian population and are consistent with studies from similar low-income settings [4,14,23–26], suggesting that the psychosocial dimensions of TB remain under-addressed in current treatment paradigms. The high prevalence of these symptoms underscores the urgent need to integrate mental health screening and support services into routine TB care, particularly given the well-documented relationship between depression and treatment adherence [27].

The mean utility score of 0.91 (±0.14) in this study population indicates a moderately high self-assessment of health status among TB patients, though still significantly lower than Ethiopian general population norms (0.94) [14]. This finding aligns with global evidence demonstrating TB’s detrimental impact on quality of life, though this study scores were notably higher than those reported in Malaysia (0.70) [26], Thailand (0.69) [6], South Africa (0.62, using the Zimbabwe value set) [4], Pakistan (0.43) [24], and Zimbabwe (0.67) [5]. This discrepancy likely reflects Ethiopia’s unique socio-cultural context and health system strengths. Culturally, Ethiopia’s strong collectivist traditions and predominant religious faith may provide robust social support networks that buffer against TB-related quality-of-life impacts a phenomenon documented in studies [28]. Systemically, Ethiopia’s free universal TB treatment eliminates financial toxicity that worsens HRQoL elsewhere [3]. Methodologically, our use of the Ethiopian value set (which may weight dimensions differently than other countries’ sets) and urban-dominated sample (97%) further caution against direct cross-national comparisons. These findings highlight how local cultural assets (social cohesion, spiritual coping) and health system achievements (accessible care) may collectively preserve HRQoL during TB treatment, while underscoring the critical need for country-specific utility values in economic evaluations [29]. Future research should explore how these protective factors might be leveraged to improve TB care models in other resource-limited settings.

The multivariable analysis identified several critical determinants of HRQoL impairment. The dramatic decline among elderly patients (≥65 years: 0.51 vs 18–24 years: 0.95) exceeds age-related declines in general populations, suggesting synergistic effects of TB and geriatric vulnerability that demand specialized clinical approaches [2,30,31]. Socioeconomic factors showed particularly strong associations, with unemployed patients demonstrating 0.23 lower utility scores than employed counterparts, a difference exceeding minimal clinically important differences for EQ-5D-5L. This economic gradient, also evident in income-stratified results (<3000 ETB: 0.86 vs > 9000 ETB: 0.99), reinforces calls for integrated socioeconomic support in TB programs [32,33].

Clinical characteristics followed expected but important patterns. The significant HRQoL reduction in HIV-co-infected patients (0.85 vs 0.92, p < 0.001) reinforces the value of integrated TB-HIV services [34]. The temporal improvement pattern (month 1: 0.82 vs month 4^+^: 0.95) provides quantitative evidence for the progressive HRQoL benefits of successful treatment [7,35,36], while highlighting the critical need for enhanced supportive care during initial treatment months when side effects and psychological adjustment are most acute. This finding suggests that interventions targeting early treatment phases could yield particularly large quality-of-life benefits.

Notably, the analysis revealed clinically meaningful gender differences despite non-significant overall utility scores (p = 0.177). This is possibly due to: comparable treatment access across genders in our setting, uniform disease severity at diagnosis, measurement properties of EQ-5D-5L in this context. However, women’s greater physical health limitations (mobility: 10.7% vs 8.8%; pain: 54.7% vs 51.8%) may reflect biological differences in pain perception or gendered care-seeking behaviors, while men’s higher anxiety/depression (56.2% vs 54.4%) aligns with emerging evidence on male psychological vulnerability in chronic illness [26]. These findings suggest TB programs should consider gender-tailored approaches while maintaining comprehensive HRQoL monitoring.

The study’s findings should be interpreted considering several limitations that could have affected the results. The cross-sectional design prevents causal inference about observed associations. Our urban sampling (97%) may limit generalizability to rural populations where healthcare access differs substantially. The EQ-5D-5L, while validated for Ethiopian use [14], may not capture certain cultural dimensions of wellbeing. Despite limitations, findings align with regional evidence. The severe HRQoL impairment in elderly and HIV co-infected patients matches South African and Zimbabwean data. However, the exploratory nature of some analyses warrants caution, only associations with p < 0.01 should be considered robust given multiple comparisons. Results best apply to urban Ethiopian TB clinics with similar DOTS programs. Findings are less generalizable to rural populations, countries without free TB treatment, or non-African settings due to cultural and structural differences. While utility values are valid for Ethiopian economic evaluations, clinical findings may extend to comparable high-burden, low-income contexts.

These findings have immediate implications for clinical practice and health policy. First, they strongly support routine HRQoL assessment in TB clinics using standardized tools. Second, they identify high-risk groups (elderly, economically disadvantaged, TB-HIV co-infection, early treatment phase) meriting targeted interventions. Third, they provide essential utility data for economic evaluations of TB interventions in Ethiopia. Future research should examine longitudinal HRQoL trajectories, test culturally adapted interventions for identified risk groups, and explore protective social factors that may inform care models elsewhere.

## Conclusion

This study reveals significant HRQoL impairments among TB patients in Ethiopia, particularly in mental health (55.4% anxiety/depression) and pain/discomfort (52.7%) domains. To address these deficits, the study recommend institutionalizing routine HRQoL assessments within Ethiopia’s TB programs using standardized tools like the EQ-5D-5L, with specific attention to high-risk groups (elderly, unemployed, and HIV-co-infected patients). The study findings provide the first Ethiopian utility values (mean = 0.91 ± 0.14) to guide cost-effectiveness evaluations and monitor progress toward the WHO’s patient-centered care targets. Integrating HRQoL metrics into clinical practice will ensure TB management addresses both biomedical and quality-of-life outcomes, a critical step for equitable care in resource-limited settings.

## Supporting information

S1 Table List of sample health centers and number of TB patients included in the study. (DOCX)

S1 File STROBE Checklist PONE-D-24–43444. (DOCX)

S2 File PLOS ONE Clinical Studies Checklist PONE-D-24–43444. (DOCX)

S3 File Raw data PONE-D-24–43444. (SAV)

## Abbreviations

EQ-5D-5L: 5-level EuroQol-5 dimensions
EQ-VAS: EuroQol Visual Analogue Scale
HRQoL: Health-related quality of life
TB: Tuberculosis
