# Supplementary material for: Health-Related Quality of Life measured with EQ-5D-5L among tuberculosis patients in Addis Ababa, Ethiopia: Institutional-based cross-sectional study
Source: PLoS One. 2025 Jun 24;20(6):e0326033. doi: 10.1371/journal.pone.0326033 (PMC12186932; doi:10.1371/journal.pone.0326033)
Supplement: S1 Table — (DOCX) [file pone.0326033.s001.docx]

**S1 Table: List of sample health centers and number of TB patients included in the study**

| Health center | Sub city | Total number of TB patients being treated | No. of TB patients included in the study |
| --- | --- | --- | --- |
| Addis Ketema Health Center | Addis Ketema Sub City | 32 | 31 |
| Addis Raey Health Center | Addis Ketema Sub City | 51 | 50 |
| Akaki Health Center | Akaki Kality Sub City | 45 | 44 |
| Gelan Health Center | Akaki Kality Sub City | 24 | 23 |
| Beata Health Center | Arada Sub City | 27 | 26 |
| Janmeda Health Center | Arada Sub City | 35 | 34 |
| Bole 17/Bole Health Center | Bole Sub City | 31 | 30 |
| Bole 17/20 Health Center | Bole Sub City | 15 | 15 |
| Shegole Health Center | Gulele Sub City | 21 | 20 |
| Shromeda Health Center | Gulele Sub City | 38 | 37 |
| Howot Amba Health Center | Kirkos Sub City | 25 | 24 |
| Efoyeta Health Center | Kirkos Sub City | 16 | 16 |
| Woreda 4 Health Center | Kolfe Subcity | 62 | 60 |
| Alem Bank Health Center | Kolfe Subcity | 41 | 40 |
| Abinet Health Center | Lideta Sub City | 28 | 28 |
| Woreda 2 Health Center | Lideta Sub City | 33 | 32 |
| Woreda 03 Health Center | Nifas Silk Lafto Sub City | 28 | 28 |
| Woreda 09 Health Center | Nifas Silk Lafto Sub City | 59 | 57 |
| Raey Health Center | Yeka Sub City | 29 | 28 |
| Woreda 13 Health Center | Yeka Sub City | 50 | 49 |
| **Total** | | **690** | **672** |
