# Supplementary material for: Health-Related Quality of Life measured with EQ-5D-5L among tuberculosis patients in Addis Ababa, Ethiopia: Institutional-based cross-sectional study
Source: PLoS One. 2025 Jun 24;20(6):e0326033. doi: 10.1371/journal.pone.0326033 (PMC12186932; doi:10.1371/journal.pone.0326033)
Supplement: S1 File — (DOCX) [file pone.0326033.s002.docx]

**STROBE Checklist**

**Title:** Health-Related Quality of Life measured with EQ-5D-5L among Tuberculosis Patients in Addis Ababa, Ethiopia: Institutional-Based Cross-Sectional Study

|  | Item No | Recommendation | Reported on page No & lines |
| --- | --- | --- | --- |
| **Title and abstract** | 1 | (*a*) Indicate the study’s design with a commonly used term in the title or the abstract | Page 1, lines 2 and 13. |
|  |  | (*b*) Provide in the abstract an informative and balanced summary of what was done and what was found | Page 1-2, lines 9-26 |
| Introduction | | |  |
| Background/rationale | 2 | Explain the scientific background and rationale for the investigation being reported | Pages 2-3\| lines 28-55 |
| Objectives | 3 | State specific objectives, including any prespecified hypotheses | Page 3\|lines 56-58 |
| Methods | | |  |
| Study design | 4 | Present key elements of study design early in the paper | Page 3\| line 63 |
| Setting | 5 | Describe the setting, locations, and relevant dates, including periods of recruitment, exposure, follow-up, and data collection | Pages 3-4\|lines 61-72 |
| Participants | 6 | (*a*) Give the eligibility criteria, and the sources and methods of selection of participants | Page 4 \|lines 81-86 |
| Variables | 7 | Clearly define all outcomes, exposures, predictors, potential confounders, and effect modifiers. Give diagnostic criteria, if applicable | N/A |
| Data sources/ measurement | 8* | For each variable of interest, give sources of data and details of methods of assessment (measurement). Describe comparability of assessment methods if there is more than one group | Page 6 \|lines 113-130 |
| Bias | 9 | Describe any efforts to address potential sources of bias | N/A |
| Study size | 10 | Explain how the study size was arrived at | Pages 4-5\|lines 88-104 |
| Quantitative variables | 11 | Explain how quantitative variables were handled in the analyses. If applicable, describe which groupings were chosen and why | Page 8/lines 155-159 |
| Statistical methods | 12 | (*a*) Describe all statistical methods, including those used to control for confounding | Page 8/lines 159-168 |
|  |  | (*b*) Describe any methods used to examine subgroups and interactions | N/A |
|  |  | (*c*) Explain how missing data were addressed | N/A |
|  |  | (*d*) If applicable, describe analytical methods taking account of sampling strategy | N/A |
|  |  | (*e*) Describe any sensitivity analyses | N/A |
| Results | | |  |
| Participants | 13* | (a) Report numbers of individuals at each stage of study—eg numbers potentially eligible, examined for eligibility, confirmed eligible, included in the study, completing follow-up, and analysed | Page 9/ line 183 |
|  |  | (b) Give reasons for non-participation at each stage | N/A |
|  |  | (c) Consider use of a flow diagram | N/A |
| Descriptive data | 14* | (a) Give characteristics of study participants (eg demographic, clinical, social) and information on exposures and potential confounders | Page 9/lines 183-189 |
|  |  | (b) Indicate number of participants with missing data for each variable of interest | N/A |
| Outcome data | 15* | Report numbers of outcome events or summary measures | Pages 9-15/lines 190-231 |
| Main results | 16 | (*a*) Give unadjusted estimates and, if applicable, confounder-adjusted estimates and their precision (eg, 95% confidence interval). Make clear which confounders were adjusted for and why they were included | Pages 15-20/lines 233-265 |
|  |  | (*b*) Report category boundaries when continuous variables were categorized | N/A |
|  |  | (*c*) If relevant, consider translating estimates of relative risk into absolute risk for a meaningful time period | N/A |
| Other analyses | 17 | Report other analyses done—eg analyses of subgroups and interactions, and sensitivity analyses | N/A |
| Discussion | | |  |
| Key results | 18 | Summarise key results with reference to study objectives | Page 20\| lines 267-270 |
| Limitations | 19 | Discuss limitations of the study, taking into account sources of potential bias or imprecision. Discuss both direction and magnitude of any potential bias | Pages 22-23 \| lines 323-327 |
| Interpretation | 20 | Give a cautious overall interpretation of results considering objectives, limitations, multiplicity of analyses, results from similar studies, and other relevant evidence | Pages 23\| lines 327-331 |
| Generalisability | 21 | Discuss the generalisability (external validity) of the study results | Page 23\|lines 331-335 |
| Other information | | |  |
| Funding | 22 | Give the source of funding and the role of the funders for the present study and, if applicable, for the original study on which the present article is based | Page 25\| line 369-370 |
