# Supplementary material for: Health-Related Quality of Life measured with EQ-5D-5L among tuberculosis patients in Addis Ababa, Ethiopia: Institutional-based cross-sectional study
Source: PLoS One. 2025 Jun 24;20(6):e0326033. doi: 10.1371/journal.pone.0326033 (PMC12186932; doi:10.1371/journal.pone.0326033)
Supplement: S2 File — (DOCX) [file pone.0326033.s003.docx]

***PLOS ONE* Clinical Studies Checklist**

**Manuscript title:** Health-Related Quality of Life measured with EQ-5D-5L among Tuberculosis Patients in Addis Ababa, Ethiopia: Institutional-Based Cross-Sectional Study

| **Complete the following if your study involved human participants or human subjects’ data. These questions should be addressed for prospective and retrospective studies.** | | |
| --- | --- | --- |
| 1. | Did you obtain ethics approval for this study?   - If yes, please upload (file type “Other”) the original approval document you received from your ethics committee. If the original document is in another language, please also provide an English translation. - **Yes, it was ethically approved. The ethical approval letter was uploaded as per requested.** |  |
| 2. | If your study involved human participants, please report in the Methods section when participants were recruited to the study.   - **The study period when the participants were recruited is reported in the study setting and design sections.** |  |
| 3. | If you are reporting a study of medical records or archived samples, please report in the Methods section the date range in which human subjects’ data/samples were collected and the date(s) when you conducted this study.   - **N/A** |  |
| 4. | Please specify in the Methods section whether authors had access to information that could identify individual participants during or after data collection.   - **It is reported in the ethical consideration section in page 4, line 77-79 that the data is sufficiently anonymized and identifiers were not used.** |  |
| 5. | If you are reporting an observational study – i.e. cohort, case-control, and cross-sectional studies – we recommend that the work is reported as per the requirements of the STROBE guidelines, and that you provide a completed STROBE checklist as a Supporting Information file with your submission.   - **STROBE checklist is Completed and uploaded** |  |
| 6. | Please ensure that the author list and Corresponding Author entered in Editorial Manager match the author list and Corresponding Author in your manuscript file.   - **We ascertained (Ensured) and we have listed and entered the authors and their affiliations correctly.** |  |
